# Chronotropic and dromotropic responses to localized glutamate microinjections in the rat nucleus ambiguus^☆^

**DOI:** 10.1016/j.brainres.2013.10.035

**Published:** 2014-01-13

**Authors:** Karla N. Sampaio, Hélder Mauad, K. Michael Spyer, Timothy W. Ford

**Affiliations:** Division of Biosciences, Faculty of Life Sciences, University College London, Gower Street, London WC1E 6BT, UK

**Keywords:** CVM, cardiac vagal motoneuron, Autonomic nervous system, Vagus nerve, Nucleus ambiguus, Heart rate, Glutamate

## Abstract

The cardioinhibitory effects of cardiac vagal motoneurons (CVMs) are mediated by activation of postganglionic neurons in the epicardial ganglia which have been shown to exert functionally selective effects on heart rate and atrioventricular conduction in the rat. Here we investigate whether CVMs producing these responses may occupy different rostrocaudal positions within the nucleus ambiguus. Excitation of CVMs was attempted by microinjections of glutamate into the nucleus ambiguus of an arterially perfused preparation in a grid extending over 2 mm in the rostrocaudal plane using the obex as a reference point. Microinjections were paired, one made during pacing to measure changes in atrioventricular conduction (P-R interval) independent of changes in heart rate and the other looking for changes in heart period (P-P interval) un-paced. Although evidence of a differential distribution was found in 7 cases, in the majority (13/20), sites producing maximal effects on both variables coincided. Maximal changes in atrioventricular conduction resulted from more rostral sites in 6 cases and from a more caudal site in only one. Overall, the ratio of the change in atrioventricular conduction to the change in heart rate for a given site was significantly greater 1 mm rostral to the obex than at either end of the test grid. We conclude that while CVMs controlling atrioventricular conduction are distributed with a peak somewhat rostral to those controlling heart rate in a number of animals, there is a significant overlap and much greater variability in this distribution in the rat than in cats and dogs.

## Highlights

• A reduced, perfused preparation of the rat heart and brain stem was employed.
• Microinjections of glutamate were made into the nucleus ambiguus.
• Resulting falls in heart rate and A–V conduction were measured from the ECG.
• A partial functional vagal efferent localization in this nucleus is described.
• Considerable inter-animal variability and overlap was found in this localization.

## 1 Introduction

The vagus carries the parasympathetic motor supply to the heart where it exerts a predominantly inhibitory action. Increased cardiac vagal activity thus results in a fall not only in the heart rate (negative chronotropic effect) but also in the rate of propagation of the cardiac action potential (negative dromotropic effect) and in the force of myocardial contraction (negative inotropic effect) (see Jones (2001)). The vagal motor pathway comprises a preganglionic component, originating in the medulla oblongata, which in turn synapses with a postganglionic component within the cardiac ganglia which are located predominantly in the atrial epicardium. Although axonal tracing studies have shown that mammalian preganglionic cardiac vagal motoneurons (CVMs) arise from the nucleus ambiguus, dorsal motor vagal nucleus and the intermediate reticular formation, the nucleus ambiguus represents the principal site of origin of myelinated cardioinhibitory B-fibers (McAllen and Spyer, 1976, McAllen and Spyer, 1978, Nosaka et al., 1979) while the dorsal motor vagal nucleus gives rise to unmyelinated C-fibers which have a lesser effect on the heart and little demonstrable input from the baroreceptors (Ford and McWilliam, 1986, Ford et al., 1990, Jones et al., 1995, Jones et al., 1998, Nosaka et al., 1982).

Earlier studies from this laboratory have shown that it is possible to produce excitation of discrete epicardial ganglia resulting in selective effects on the rat heart (Sampaio et al., 2003). These data are consistent with the existence of functionally selective ganglia that have been demonstrated in other species (Ardell and Randall, 1986, Gatti et al., 1995, Gatti et al., 1997, Randall et al., 1986a, Randall et al., 1986b).

In the cat, the vagal preganglionic axons running from the nucleus ambiguus to the heart appear to target specific ganglionic clusters and therefore may also be functionally defined at the preganglionic level (Gatti et al., 1996, Massari et al., 1995). That preganglionic CVMs in the rat nucleus ambiguus may also be functionally specific is suggested by the results of preliminary tracing studies performed in this laboratory. Single discrete microinjections of anterograde tracers that label small numbers of vagal preganglionic motoneurons in the nucleus ambiguus result in a restricted distribution of labeled axon terminals in the cardiac ganglia (Jones et al., 1999). While precise mapping of the injection sites to anatomically identified ganglia was not performed in this study, labeled axons were traced running through ganglionated plexuses without making any apparent synaptic contact until reaching an apparent target zone where dense innervation of neighboring ganglion cells occurred. This suggests that single preganglionic CVMs may preferentially innervate ganglion cells projecting to one particular target rather than multiple sites within the heart. That such a targeting exists is supported by anterograde tracing experiments which have shown that CVMs from the left and right sides of the brain stem appear to innervate different subdivisions within the cardiac ganglionic plexuses (Cheng et al., 2004).

In the study by Gatti et al. (1996), sites in the nucleus ambiguus producing negative dromotropic effects to glutamate tended to be located rostral to those producing negative chronotropic effects. In some rostral locations, sites producing dromotropic responses with no effects on heart rate were reported. The present experiments investigate whether differential cardiac effects can be elicited by focal application of glutamate along the rostrocaudal extent of the rat nucleus ambiguus while monitoring the effects on heart rate and atrioventricular conduction.

A preliminary report has been published in abstract form (Sampaio et al., 2000).

## 2 Results

### 2.1 Effects of microinjections of glutamate

**Fig. 1:**
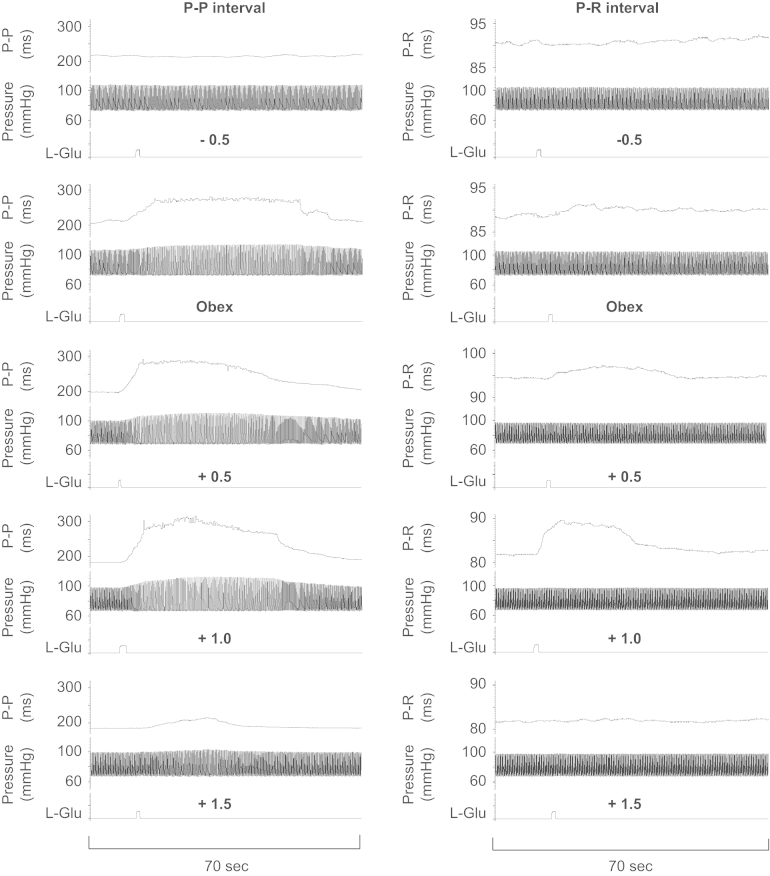
Examples of Spike2 traces showing the changes in P-P and P-R intervals following glutamate microinjections at 5 levels of the right nucleus ambiguus in one preparation. Recordings of P-R intervals were performed during cardiac pacing. Note that changes in heart rate (P-P interval) had relatively little effect on the perfusion pressure which was largely determined by the flow rate set by the perfusion pump less the flow through the bypass circuit. The pulse pressure arises through the addition to the perfusate of the contents of the left ventricle, the filling of which was not controlled for in these experiments i.e. no attempt was made to eliminate return from pulmonary veins. There was a relatively small effect on pulse pressure presumably attributable to greater diastolic filling and therefore greater ejection through the Frank–Starling mechanism.

Glutamate microinjections commonly elicited changes in heart rate and atrioventricular conduction when the electrode tip was between 1.5 and 1.8 mm lateral to the midline and at a depth of 1.6–1.9 mm depending on the rostrocaudal level relative to the obex. A representative example of the time course and extent of changes in P-P and P-R intervals at different rostrocaudal levels is illustrated in Fig. 1.

Responses typically occurred with an onset latency of less than 5 s, peaked some 5–30 s later and returned to baseline levels within 1–2 min. The responses were reproducible within any series of injections and could not be elicited by injections of equivalent volumes of vehicle (20 nL Ringer's solution). The sizes of the responses varied quite considerably however from one preparation to another. Sites producing maximal responses were well-localized; microinjections 0.5–1.0 mm medial or lateral to these sites were typically ineffective or produced comparatively small responses at longer onset latencies. Significant increases in both P-P and P-R interval were observed following microinjection of glutamate at all levels of the nucleus ambiguus studied when compared to pre-injection levels (Table 1).

**Table 1:** Baseline and maximum responses to glutamate injection into the nucleus ambiguus at the levels shown.

A	Level of injection	−0.5 (*n*=13)	0 (*n*=19)	+0.5 (*n*=10)	+1.0 (*n*=19)	+1.5 (*n*=16)
	Pre-injection P-P (ms)	232±5.6	232±6.0	207±4.5	228±5.4	236±6.1
	Post-injection P-P (ms)	244±6.8⁎	289±12⁎⁎	267±14⁎⁎	268±10⁎⁎	247±6.1⁎⁎

B	Level of injection	−0.5 (*n*=12)	0 (*n*=19)	+0.5 (*n*=10)	+1.0 (*n*=19)	+1.5 (*n*=16)
	Pre-injection P-R (ms)	76±3.9	76±3.0	78±4.8	78±3.4	74±3.1
	Post-injection P-R (ms)	77±3.8⁎	83±3.5⁎⁎	84±5.4⁎⁎	87±4.5⁎⁎	76±3.1⁎

Values represent the mean±SEM.

For the level of injection, 0 represents the level of the obex with + being rostral and – being caudal relative to obex.

Note that the availability of paired data allowed the detection of significant differences despite the relatively small size of the responses and the high variability indicated by the SEMs.

⁎⁎ *p*<0.01.

⁎ *p*<0.05 significantly different from control using a *paired* t-test, two-tailed.

### 2.2 Rostrocaudal distribution

**Fig. 2:**
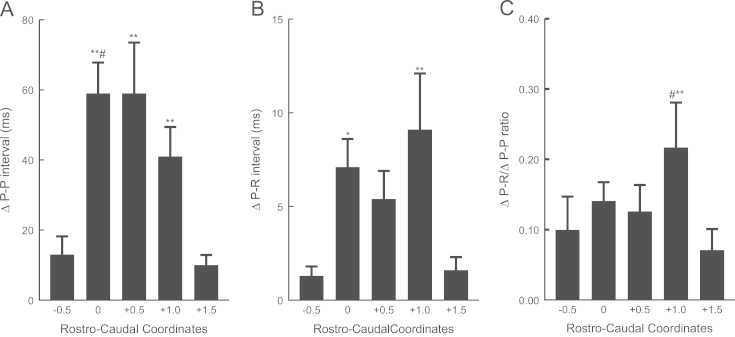
Changes in P-P interval (A), P-R interval (B) and ∆P-R/∆P-P (C) following microinjections of glutamate at 5 levels of the NA. In (A), ^⁎⁎^*p*<0.01 indicates statistically significant differences compared to the −0.5 and +1.5 level and #*p*<0.05 indicates a statistically significant difference compared to the +1.0 level. In (B), ^⁎⁎^*p*<0.01 and ^⁎^*p*<0.05 indicate statistically significant differences compared to −0.5 and +1.5 levels. In (C), #*p*<0.05 and ^⁎^*p*<0.01 indicate statistically significant differences compared to the −0.5 and +1.5 levels, respectively. The distribution of sites affecting P-P interval (heart rate) is unimodal, peaking between obex and 0.5 mm rostral (A). The distribution of sites affecting P-R interval are more skewed in the rostral direction with the largest peak at 1 mm rostral to obex (B). The site producing the greatest change in P-R interval per unit change in P-P interval is at 1 mm rostral to obex (C).

For each injection level the overall change in P-P interval (unpaced) is illustrated in Fig. 2A. Comparing the effectiveness of different sites in eliciting changes in P-P interval it can be seen that microinjection of glutamate at 0, +0.5 and +1 induced significantly greater changes in P-P interval compared to the other two sites. Changes in P-R interval however (Fig. 2B) were observed to be significantly greater only at the levels 0 and +1.0 compared to the changes elicited at the most rostral and most caudal sites. To compare the relative effectiveness of microinjections on chronotropic and dromotropic responses, the ratio of the change in P-P interval to the change in P-R interval (∆P-R/∆P-P) was calculated for all sites where both measurements were made (i.e. microinjections during both unpaced and paced runs). The intention was to avoid loss of valuable paired data which would otherwise occur when data were pooled. If the CVMs controlling P-P interval and those controlling P-R interval were mixed homogeneously across the rostrocaudal extent of the nucleus then one would not expect to find peaks in this ratio at any particular rostrocaudal location.

In the event, ∆P-R/∆P-P peaked at 1 mm rostral to obex which reached significance when compared to −0.5 and +1.5 but not to 0 and +0.5 (Fig. 2C).

**Fig. 3:**
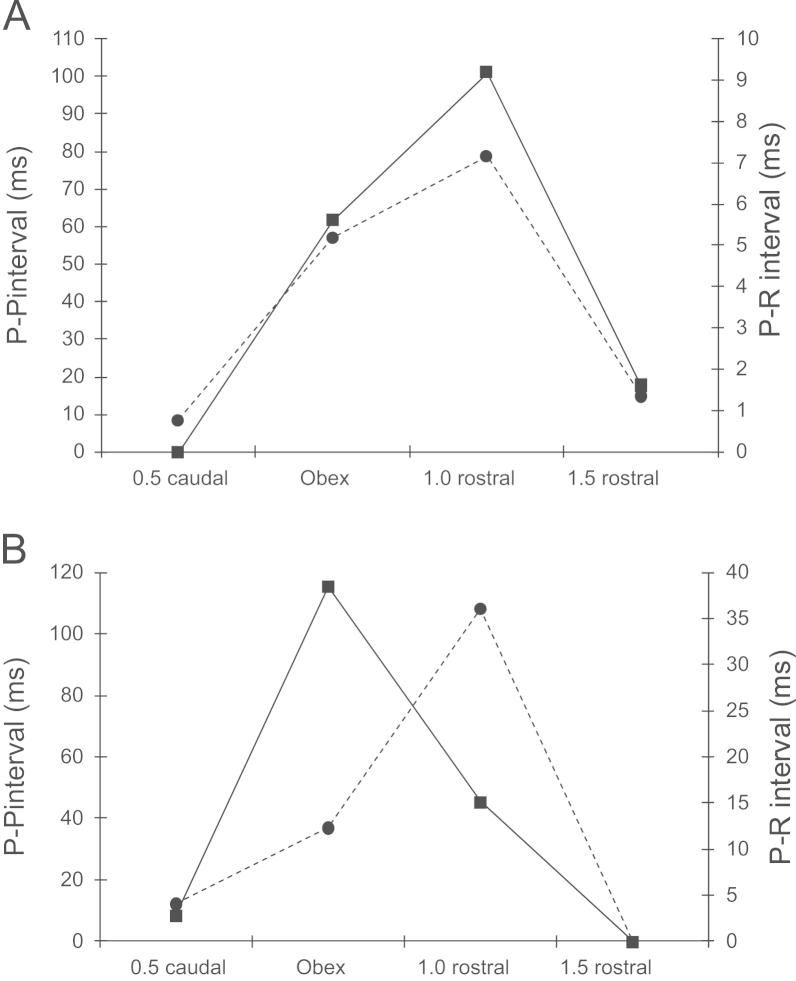
Responses to glutamate microinjections into the right nucleus ambiguus on changes in P-P interval (left axis, squares with solid lines) and P-R interval (right axis, circles with broken lines) in two preparations. In (A), the size of the response on one variable follows that on the other, both peaking 1 mm rostral to obex. In (B), there is a clear separation of the peaks with a maximal change in P-R interval 1 mm rostral to obex while the peak change in P-P interval occurs at obex. This suggests that in this preparation (B) CVMs affecting atrioventricular conduction were localized at +1 compared to obex where there was a preponderance of CVMs affecting heart rate. Note the differences in the PR axis scales in (A) and (B) illustrating the variability in the sizes of the responses obtained in different preparations.

**Fig. 4:**
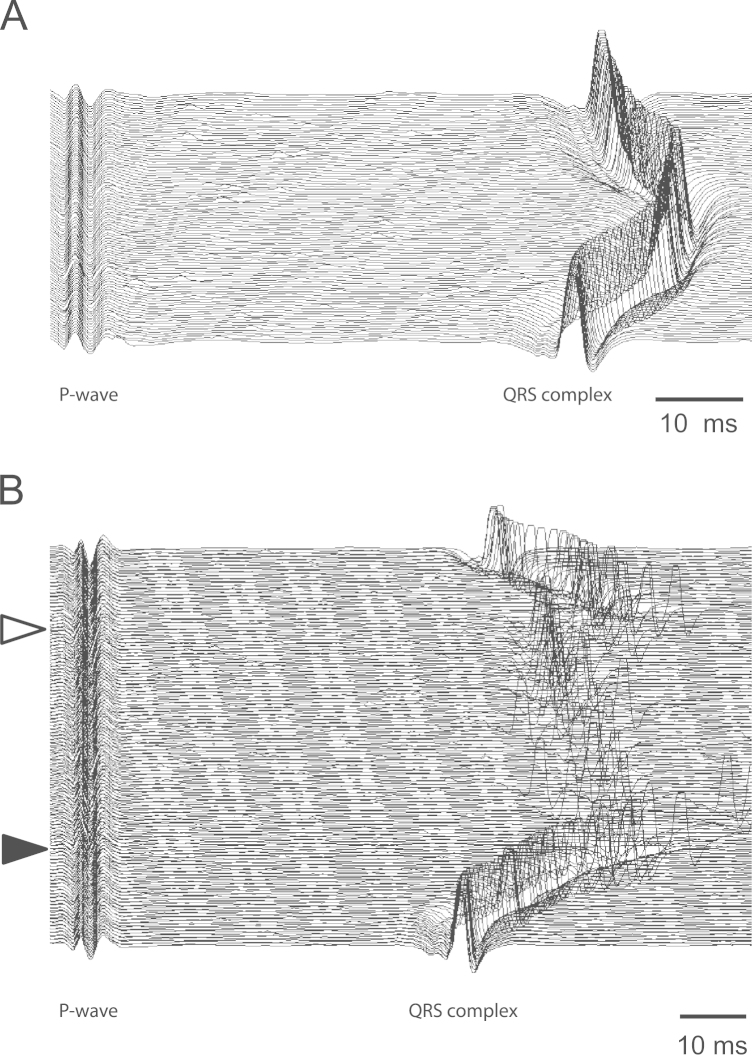
Waterfall plots showing beat-by-beat changes in P-R interval following microinjections into the nucleus ambiguus (A) at the level of the obex and (B) 1.0 mm rostral to the obex. In both traces, the atria were paced at a rate slightly above the resting heart rate. Glutamate was injected just prior to the first sweep which is at the bottom in both plots with subsequent sweeps displaced by a small increment in the *Y*-axis. The time between the start of each sweep, which corresponds to the P wave and the start of the QRS complex (i.e. the PR interval) can be seen to lengthen in both cases. In (B) however, second degree atrioventricular block can be seen to develop from the sweep indicated by the dark arrowhead on the left. In this condition, one or more of the cardiac action potentials fails to be conducted across the atrioventricular node. This condition remains until the sweep indicated by the clear arrowhead at which point all action potentials begin once again to reach the ventricles. Between the arrows, a variable number of action potentials are successfully conducted to the ventricles, typically at a reduced P-R interval presumably attributable to the removal of the refractory effects of the non-conducted action potential.

The distribution of the peaks in these histograms is skewed in the caudal direction in A compared to B and C suggesting a greater preponderance of chronotropic CVMs at caudal than at rostral sites. The tendency for the more rostral sites to have a greater effect on P-R than on P-P is also apparent from the relative positions of the most effective sites in any set of unilateral microinjections in individual preparations: most (13/20) showed peak changes in both P-P and P-R interval following injections at the same level (see example in Fig. 3A). However in 6/20, peak changes in P-R were elicited from sites rostral to those eliciting peak changes in P-P (see example in Fig. 3B) and in only 1 was the reverse found. At 9 sites in 8 preparations atrioventricular conduction block was observed following glutamate microinjections between obex and 1 mm rostral to obex but not below (see example in Fig. 4B).

### 2.3 Left versus right

**Fig. 5:**
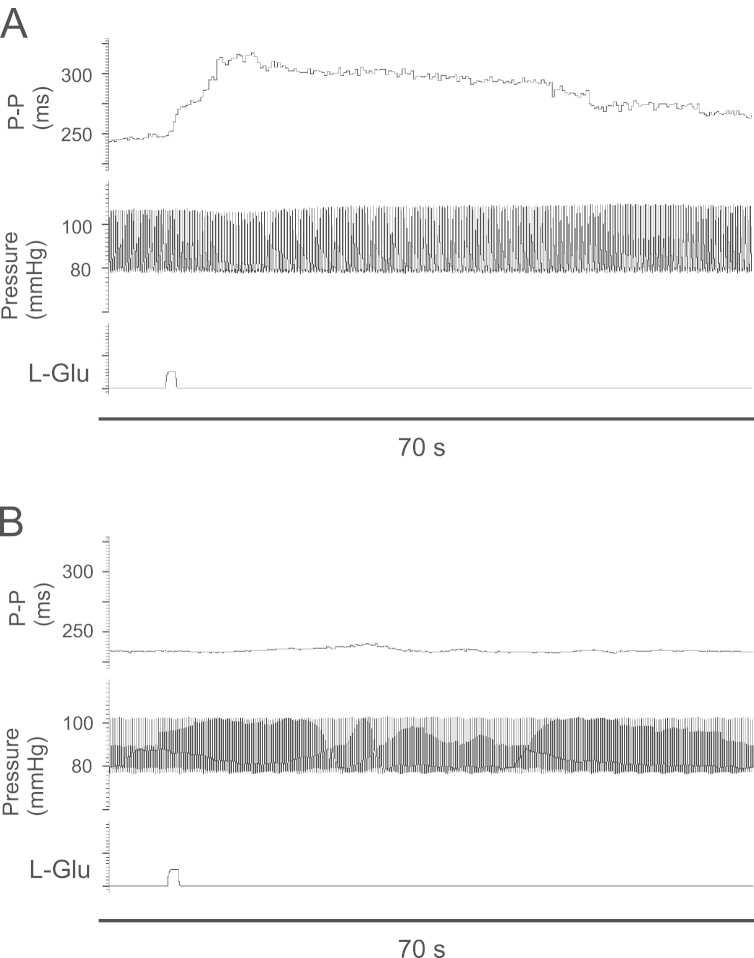
Examples of Spike2 traces showing the changes in P-P interval following glutamate microinjections into the nucleus ambiguus (A) before and (B) after ipsilateral vagal section in one preparation. Vagal section abolished the characteristic short-latency bradycardia evoked by glutamate. The small, transient bradycardia seen at about 20 s in (B) was not reproducible and most likely represents a spontaneous minor variation in the heart rate in this case. Since the ipsilateral vagus was cut and the spinal cord sectioned, activation of pathways to the contralateral vagal motor nuclei would be the only remaining possibility.

Microinjections of glutamate into the left nucleus ambiguus were statistically no more or less effective than those on the right at prolonging P-P or P-R interval (*p*>0.05). However, it is worth noting that 6 out of the 9 sites producing atrioventricular block were on the left. This is of relevance because when second degree atrioventricular block intervened, the preceding P-R interval was usually taken as the maximum size of the response. In two animals an ipsilateral vagotomy was performed which abolished the typical response induced by glutamate injections into the nucleus ambiguus (Fig. 5). Stimulation of the peripheral cut ends of the sectioned vagi elicited significant effects on both P-P and P-R interval and did not appear to show any right/left selectivity on either.

**Fig. 6:**
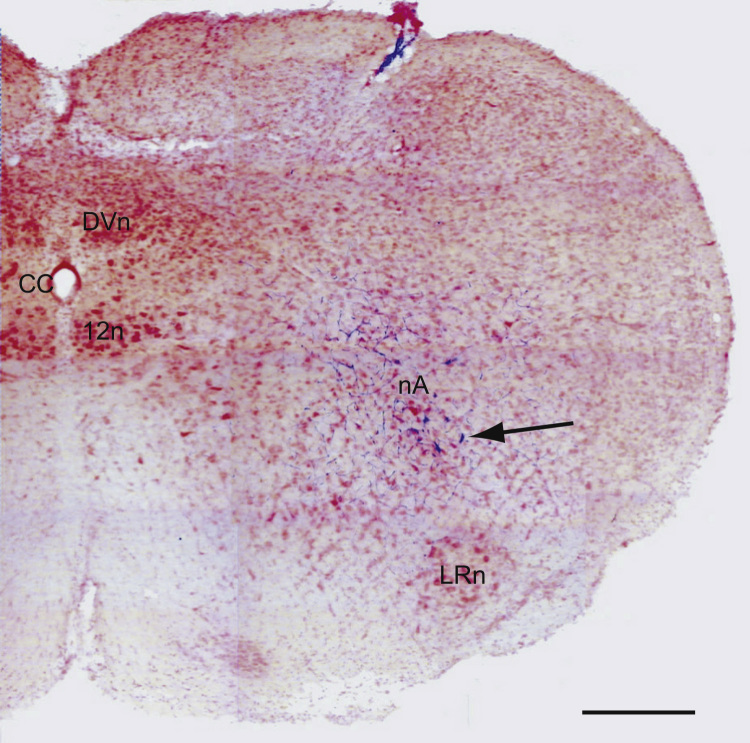
Photomontage of a transverse section from the brain stem stained with neutral red showing biocytin-labeled neurons (blue, arrowed) following a microinjection in the right nucleus ambiguus of one animal (kp11). 20 nL biocytin 2% in 0.5 M NaCl was injected at a point 0.5 mm caudal to obex, 1.6 mm lateral to the midline and at a depth of 1.6 mm. Scale bar 500 µm. The nucleus ambiguus at this level is indistinct and CVMs are found outside the main column (Corbett et al., 2007, Kalia and Sullivan, 1982). Note the absence of labeling in the lateral reticular nucleus (LRn). nA – nucleus ambiguus, DVn – dorsal vagal nucleus, 12n – hypoglossal nucleus.

Sites eliciting rapid-onset responses that were marked with biocytin were shown to be localized within the area of the nucleus ambiguus when visualized under a conventional light microscope. An example of biocytin labeled cells in the area of the right nucleus ambiguus following a microinjection at −0.5 mm relative to obex is reproduced in Fig. 6. In this case, the biocytin was pressure injected to a volume of approximately 20 nL and resulted in labeled cells within the area of the nucleus ambiguus and the surrounding periambigual regions but not in the adjacent lateral reticular nucleus situated about 0.5 mm ventrolateral to it (Fig. 6).

## 3 Discussion

If preganglionic cardiac vagal motoneurons innervated their target neurons in the cardiac ganglia in a non-selective manner and irrespective of their location in the medulla oblongata, then one would expect to see homogeneity in the responses evoked by their excitation at different rostrocaudal levels. So preganglionic neurons in the rostral extent of their distribution would be expected to elicit similar responses on heart rate and the rate of atrioventricular conduction as neurons found toward the caudal pole. The results of the present study have shown that there is in fact a considerable degree of variation in the responses evoked at different rostrocaudal levels. This is illustrated in Fig. 3 where a comparatively homogeneous response in (A) is contrasted with that in (B) where a much greater effect was elicited on heart rate (as reflected in the P-P interval) than on atrioventricular conduction (as reflected by the P-R interval) at the level of the obex and was reversed just 1 mm rostral. This suggests that at least in some animals, there is a spatial separation of preganglionic motoneurones innervating the different cardiac ganglia which have been shown to exert such differential effects in this species (Sampaio et al., 2003). Where a differential effect was observed, the sites eliciting maximal effects on atrioventricular conduction were rostral to those eliciting maximal effects on heart rate in all but one case (6/7). Such a “cardiotopic” organization of the nucleus ambiguus has been previously described in the cat and dog (Blinder et al., 1998, Blinder et al., 2007, Gatti et al., 1996, Massari et al., 1995). However, it is clear that a degree of variation exists in the topographical organization of the preganglionic neurons in the rat since in most cases (13/20) the maximal effects both on atrioventricular conduction and heart rate were obtained by microinjections at the same rostrocaudal level. The greater degree of apparent overlap in the present study may have resulted to an extent from methodological limitations since the absolute size of the nucleus containing CVMs is smaller in the rat in relation to the size of the microinjection. Central recording studies in the cat have shown that excitation of CVMs using ionophoresis of excitatory amino acids can produce measurable changes in heart rate (McAllen and Spyer, 1978), so it is possible that more discrete, low volume microinjections (Shaw et al., 2002) or ionophoretic application of glutamate would have revealed a clearer topographical localization. However, the fact that quite distinct differential effects could be elicited in individual preparations suggests that methodology was not the primary reason for the variability in the relative positions of chronotropic and dromotropic maxima. Rather it seems more likely that there exists a significant variability from animal to animal in the extent of the localization and the degree of overlap. This may also reflect an imperfect segregation apparent in the functional characteristics of the cardiac vagal ganglia in individual rats where selective electrical stimulation of the ganglia was attempted (Sampaio et al., 2003).

The conclusion is based upon the assumption that the microinjections of glutamate produced their effects primarily through direct activation of CVMs in the area of the nucleus ambiguus and that the same subset of CVMs were excited by repeat microinjections into the same area. The technique was chosen in preference to electrical stimulation to avoid activation of axons of passage which are known to pass through this area from other regions of the brain stem e.g. dorsal motor vagal nucleus and nucleus tractus solitarius, (Kalia and Mesulam, 1980, Kalia and Sullivan, 1982). Glutamate is known to excite neurons while leaving axons of passage unaffected (Goodchild et al., 1982, Zieglgansberger and Puil, 1973) and CVMs express glutamate receptors of both AMPA and NMDA subtypes (Corbett et al., 2003) but it is possible that local circuits were activated as well as CVMs. However, support for the assumption is provided by the observation that effects were rapid in onset and strongly localized. Injections outside the immediate area of the nucleus were smaller and had much longer onset latencies. Furthermore the rapid onset cardiac effects were abolished by ipsilateral vagal section (Fig. 5). Since the spinal cord was sectioned at C2, sympathetically mediated effects are unlikely to be operational, leaving synaptic activation of contralateral CVMs as the only likely remaining neuroeffector mechanism. While the small, brief bradycardia seen in Fig. 5B about 20 s post-injection may be attributable to such a mechanism rather than being a random occurrence, it is considered unlikely that this would have made a significant contribution to the measured responses. Attempts to block synaptically-evoked responses in the brain stem using a low-calcium, high-magnesium Ringer's solution were unsuccessful since synaptic blockade also occurred at the ganglia despite efforts to selectively perfuse the coronary arteries with the normal Ringer's solution. This was attributed to a significant vascular supply to the cardiac ganglia via an extracoronary source (Halpern, 1957, Jones, 2001).

These data support the results of preliminary anterograde tracing studies following discrete injections into the nucleus ambiguus which suggest targeting by CVMs of distinct groups of cardiac ganglion cells (Jones et al., 1999). Anterograde tracing studies in the rat (Ai et al., 2007, Cheng et al., 1999, Cheng et al., 2004, Cheng and Powley, 2000) have shown in exquisite detail that both the nucleus ambiguus and the dorsal motor vagal nucleus project to all the cardiac ganglia in this species. Since CVMs from the two sites appear to target separate subpopulations of principal neurons (Cheng et al., 2004) there is an anatomical basis for differential control of cardiac function by location. The injections of anterograde tracers in the above studies were made with the aim of labeling each nucleus in its entirety rather than targeting discrete regions of the nucleus ambiguus or dorsal motor vagal nucleus so it is difficult to speculate about differential organization within any one nucleus. However, neurons in the dorsal motor vagal nucleus did not appear to converge on the same population of principal cells in the rat cardiac ganglia that were innervated by neurons of the nucleus ambiguus (Cheng et al., 2004).

Recordings made in a reduced preparation similar to that described here provide compelling evidence that a typical active principal cardiac ganglion cell receives its input from a single active CVM (McAllen et al., 2011). Furthermore, the same active CVM appears to drive reflex responses from diverse sources such as the peripheral chemoreceptors, the arterial baroreceptors and the diving response. The authors do not exclude the possibility of convergence from different CVMs. Indeed they show that convergence from silent fibers is revealed during direct electrical stimulation of the vagus but the source of the CVMs remains unknown. Silent CVMs unresponsive to reflex inputs such as those above have been described in the nucleus ambiguus and dorsal motor vagal nucleus in cats (Ford et al., 1990, McAllen and Spyer, 1978) and in rats (Jones, 2001, Rentero et al., 2002) and while they remain a source of speculation with regard to the circumstances under which they might be normally be activated, they provide little insight into whether their projections might be functionally specific. The reflex inputs described above are known to produce both chronotropic and dromotropic responses as are those resulting from stimulation of cardiopulmonary afferent fibers so the reasons for the development of separate populations of CVMs controlling heart rate and atrioventricular conduction are not immediately apparent. The suggestion that CVMs from the dorsal motor vagal nucleus might be selectively activated by cardiopulmonary inputs (Daly and Kirkman, 1989, Jones and Daly, 1997) no longer appears tenable (Jones, 2001, Wang et al., 2000). The bradycardia evoked by stimulation of cardiopulmonary afferents in the rat is attenuated after bilateral microinjection of P2X antagonist in the nucleus ambiguus suggesting CVMs in this region are also activated by this input via release of purines (Passamani et al., 2011). Interestingly, an essential role for CVMs in the dorsal motor vagal nucleus has recently been proposed for the cardioprotective effects of remote ischemic preconditioning in this species (Mastitskaya et al., 2012) which appears to be independent of effects on heart rate. The specific groups of ganglion cells mediating these effects remain to be established.

Effects on cardiac inotropy were not investigated in the present study and it has not yet been determined whether reports of a similar functional separation at the preganglionic level in the nucleus ambiguus of the cat and dog (Blinder et al., 1998, Blinder et al., 2007) are also to be found in the rat. A study looking at the release of acetylcholine in the left ventricular myocardium following vagal stimulation in the cat suggests that acetylcholine levels in the left ventricle are affected by both right and left vagal nerves and show little evidence of interactions at the level of the cardiac ganglia (Akiyama and Yamazaki, 2001). There was a great deal of variability between preparations on the relative effects of left versus right vagal nerve stimulation in the current experiments with the result that no statistically significant difference was found between the two sides with regard to effects on heart rate and atrioventricular conduction. The fact that atrioventricular block was more commonly obtained from the left nucleus ambiguus may suggest a greater involvement of the left vagus in atrioventricular conduction, as has been reported by others (Hamlin and Smith, 1968, Parker et al., 1984). Retrograde labeling of CVMs in the rat nucleus ambiguus from the cardiac ganglion nearest to the sinoatrial node has been reported to be greater on the right than the left (Corbett et al., 2007) though the authors are cautious about attributing this to a functional separation rather than a simple topographical arrangement. Similarly, anterograde labeling of CVMs in the left and right sides of the brain stem has shown that their axons enter the ganglia via different routes and project to different neuronal subdivisions within the cardiac ganglia (Cheng et al., 2004). While these findings lend support to the notion of a functional topographical arrangement of CVMs in this species, localized microinjections of anterograde tracers into different regions of the nucleus ambiguus would be required to confirm that an anatomical substrate exists for the functional organization within this nucleus. Such studies and others using retrograde tracing techniques which lay beyond the scope of the present experiments may determine whether differences exist in the mediolateral and dorsoventral localization within this nucleus in addition to those demonstrated in the rostrocaudal plane in the present experiments.

## 4 Experimental procedures

### 4.1 Preparation

Experiments were performed using a rat preparation perfused with oxygenated Ringer's solution (Ford and Spyer, 1999, Paton, 1996). All the experiments were performed in accordance with the European Commission Directive 86/609/EEC (European Convention for the Protection of Vertebrate Animals used for Experimental and Other Scientific Purposes) and the UK Home Office (Scientific Procedures) Act (1986) under institutional guidelines and every effort was made to minimize animal discomfort and numbers used. Twenty-eight juvenile Sprague-Dawley rats weighing between 100 and 150 g were deeply anaesthetized with isoflurane (5% in oxygen) delivered via a vaporizer (Fluotec 3) in a custom designed induction chamber until breathing ceased and a complete loss of withdrawal to forepaw pinch was observed. They were immediately removed from the chamber, bisected at level of the diaphragm and immersed in oxygenated Ringer's solution at 2–4 °C. The preparation was decerebrated by removal of all brain tissue above the inferior border of the colliculus using a suction tube and most of the skin removed from the neck down. The forelimbs were excised below the shoulder, following ligation of the brachial arteries, to facilitate access to the vagi and the dorsal upper thoracic cavity. The descending aorta was then cannulated and perfused retrogradely with Ringer's solution at 31-32 °C using a peristaltic pump (Watson Marlow, Cornwall, UK; flow rate, 40-60 ml min^−1^) so that the remaining tissue, including the brain stem remained viable.

**Fig. 7:**
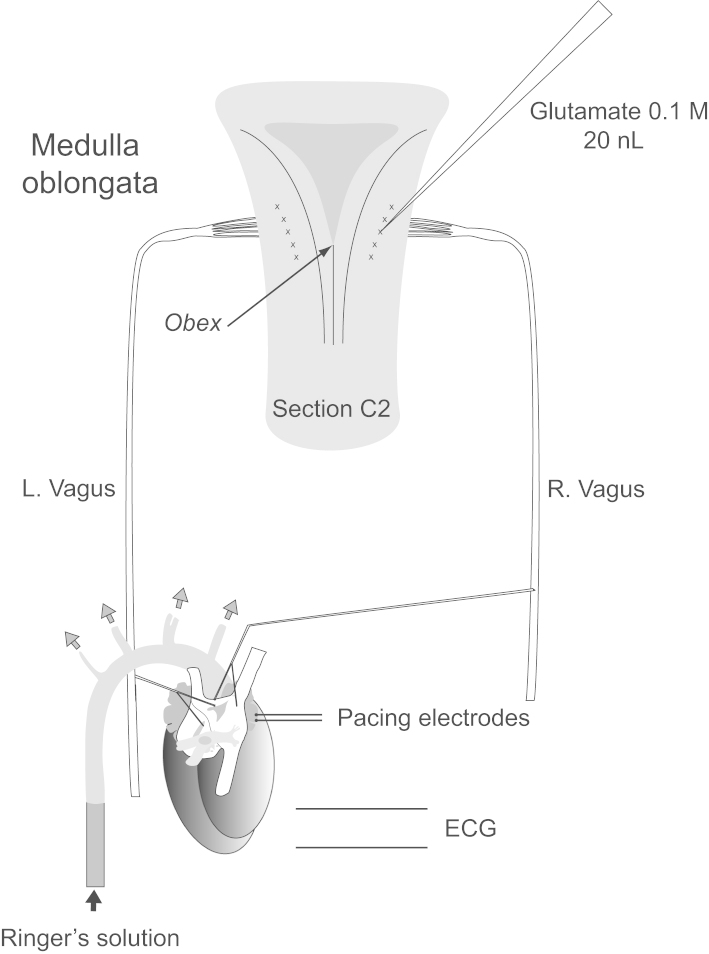
Schematic of experimental preparation. Microinjections were made according to a grid which used as its reference point the obex as illustrated in Paxinos and Watson (2007). The nucleus ambiguus moves laterally as it ascends in the rostral direction and this was taken into account in the penetrations which were all perpendicular to the dorsal surface. The ventroflexion of the head was such that the dorsal surface of the brain stem over the area explored was close to the horizontal.

The composition of the Ringer's solution was as described by Paton (1996) and contained (mM): NaCl 125, NaHCO_3_ 24, KCl 5, CaCl_2_ 2.5, MgSO_4_ 1.25, KH_2_PO_4_ 1.25 and d-glucose 20; the solution was bubbled with a mixture of 95% oxygen and 5% carbon dioxide (carbogen). Ficoll 70 (5%, Sigma) was added to the vascular perfusate to increase colloid osmotic pressure and the pH was adjusted to 7.3-7.4. Perfusion pressure was monitored using a pressure transducer (Neurolog NL108T1, Digitimer Limited, UK) attached to the minor lumen of a double lumen cannula used for the perfusion (modified from a Swan-Ganz 123F6 double lumen catheter, Baxter Healthcare, Deerfield, IL, USA) and maintained between 70 and 120 mmHg by dividing the flow appropriately through the cannula and a bypass circuit that returned perfusate directly back to the reservoir via a variable roller clamp or Hoffman clip. The preparation was held in the prone position with the head somewhat ventroflexed using an acrylic holder with ear bars and the spinal segments and ribs below the level of T4 were removed to allow access to the heart. Both cervical vagi were identified and loose threads were passed behind them to aid later placement on stimulating electrodes if required. The spinal cord was sectioned at C2 to eliminate bulbospinal sympathetic pathways (Fig. 7).

### 4.2 ECG recording and electrical pacing

The ECG was recorded using local platinum electrodes positioned close to the atria so as to give a prominent P wave as well as an identifiable QRS complex. An intelligent computer interface (CED1401plus, Cambridge Electronic Design, Cambridge, UK) was used to digitize the recordings of the perfusion pressure, ECG and stimulus event timings. The ECG signal was band-pass filtered from 10 Hz to 2 kHz and sampled at 5 kHz. Data were transferred to a PC for on-line display and held on the computer hard disk for later analysis and backup to CD-ROM. Preliminary on-line analysis of heart period (P-P interval) was achieved by level triggering from the P wave of the ECG using a window discriminator (Neurolog NL201, Digitimer Limited) which fed event time data into the 1401plus for continuous display using Spike2 software (Cambridge Electronic Design). A falling heart rate was indicated by an increase in the P-P interval of the ECG. Atrioventricular conduction time was taken as an index of dromotropy and was derived from the P-R interval. To avoid indirect effects of changes in heart rate on the P-R interval, standard measurements were undertaken during atrial pacing at constant rate. Pacing electrodes made from fine lacquered copper wire were attached to the right atrial appendage by piercing its lateral border with the bare copper wire and forming a loop. P-R interval during atrial pacing was monitored directly on an oscilloscope triggered from a timing device (Digitimer 4030) which was also used to trigger the stimulators driving the pacing and stimulating electrodes (Digitimer DS2 constant voltage isolated stimulators). The stimulus interval for pacing was set between 90% and 99% of the minimum unpaced P-P interval in the period prior to stimulation.

### 4.3 Microinjections into the NA

Pressure injections of 0.1 M l-Glutamate (20 nL in Ringer's solution) were made into the nucleus ambiguus from one barrel of a triple-barreled micropipette (20–25 µm tip) at sites from 0.5 mm caudal to 1.5 mm rostral to the obex (−0.5, 0, +0.5, +1, +1.5) using compressed nitrogen controlled by a solenoid valve (Neurophore system, Digitimer Limited). The remaining two barrels contained vehicle (Ringer's solution alone) and a biotinylated marker respectively. At each level, sites were sought that elicited maximal effects at the shortest latencies (Fig. 7). All measurements were made relative to the obex as it is described in Paxinos and Watson (2007). It is worth noting that these authors designate the obex as the caudal end of the area postrema in the rat, corresponding to the tip of the calamus scriptorius. In this preparation, this lay approximately 0.6 mm caudal to the rostral border of the area postrema at the midline which some authors identify as the obex (e.g. Kalia and Sullivan, 1982).

Microinjections commonly elicited changes in heart rate when the electrode tip was between 1.5 and 1.8 mm lateral to the midline and at a depth of 1.6–1.9 mm from the dorsal surface as measured using micromanipulators (Narishige, Japan). The mediolateral and dorsoventral coordinates of the target injection sites were initially based on histological material from an earlier series of studies but were modified on the basis of the responses obtained. Typically tracks within 0.5 mm rostral and 0.5 mm caudal to obex were selected for the first injection site and were assessed by the latency and size of the response. If a site did not elicit short latency changes in heart rate or atrioventricular conduction then the electrode was moved to a different depth or if necessary medially or laterally until optimal responses were obtained. The lateral excursion and depth increased as the brain stem was explored from caudal to rostral. The order in which the sites were explored varied but injections at the level of the obex were generally the first to be made; one side (left or right) was usually chosen for completion first. Only responses with an onset latency of 10 s or less were included in the data. After advancing the electrode to the injection site, at least two microinjections were made at each locus: one without pacing and the other during atrial pacing, to measure chronotropic and dromotropic effects, respectively. Periods of at least 2 min were allowed between microinjections to enable full recovery. All measurements of P-R interval used to assess atrioventricular conduction were made during pacing to eliminate heart rate dependent changes in this variable. The volume injected (20 nL) was measured by direct observation of the drop of the meniscus against a known scale fabricated from nylon mesh glued to the micropipette, as observed under a dissecting microscope. Control microinjections of Ringer's solution into selected sites were also made. If time permitted and the preparation remained in good condition, the contralateral brain stem was explored at the same levels. There was a tendency for edema to develop in these preparation, despite the presence of the oncotic agents in the perfusate so the condition of the preparation was tested at intervals by observing the characteristic bradycardia in response to baroreceptor stimulation (brief elevations in perfusion pressure brought about by restricting the bypass flow) or chemoreceptor stimulation (10–50 µL bolus of 0.05% sodium cyanide injected into the perfusion cannula). Reproducible responses could be maintained for at least 4 h after the initial preparation but typically measurements were completed within 3 h of the first microinjection.

### 4.4 Identification of sites of injection

A selection of injection sites were marked by the iontophoretic application (2 µA, 7 s on /7 s off, for 5 min) of biocytin (Molecular Probes) or Neurobiotin (Vector Labs UK, both 2% in 0.5 M NaCl) from the micropipette. Occasionally, the application was by pressure injection of 20 nL in an attempt to gauge injectate spread (e.g. Fig. 6). The brain stem was removed and fixed in fresh phosphate-buffered formal saline (4% paraformaldehyde in 0.1 M phosphate buffer, 0.9% NaCl, pH 7.4) for 24-72 h. Brain stems were later transferred to a sucrose solution (sucrose 10% in 0.1 M phosphate buffer) at 4 °C at least 24 h prior to sectioning. Sections were cut at 50 µm on a freezing microtome, washed twice in phosphate buffered saline (0.01 M) and incubated in the avidin–biotin–HRP complex (ABC, Vector Labs, UK). The sections were then washed and processed using diaminobenzidine (DAB, Sigma, UK) with nickel intensification. Sections were mounted on gelatin coated slides and counterstained with neutral red (1%, Sigma, UK) to visualize the marked sites. The positions of the injection sites revealed by the characteristic blue/black staining were captured by photomicrographs obtained using a Leica DMR microscope under conventional bright field illumination with a Nikon Coolpix 4500 digital camera.

### 4.5 Data analysis

The ECG data were analyzed off-line using Spike2 program scripts to rewrite the P-P and P-R intervals as waveform data following identification of ECG waves using the template matching functions of the program. All template-generated event identities were verified by visual inspection of the record. Event times were not adjusted to correspond exactly with the start of the respective waves but the trigger point for each wave was maintained during the period of measurement. In most cases during pacing, the pacing stimulus event time was used as the trigger point for the P wave. If second degree atrioventricular block developed this was noted but the P-R interval was not updated so that only fully conducted cardiac action potentials were used to derive the maximum interval. Values are expressed as the mean±standard error of the mean (S.E.M.). Statistical comparisons between baseline and maximal values of the P-P and P-R interval data at the various sites were made using Student's t test for paired data (two-tailed). Comparisons between the responses at the various levels were by one-way analysis of variance (ANOVA) followed by a Fisher test for multiple comparisons. A probability level of less than 0.05 was taken to indicate statistical significance.
